# Schistosoma mansoni-related periportal fibrosis; can we use APRI and PSDR levels in the real-time selection of patients for targeted endoscopy in a resource-limited setting? A case–control study

**DOI:** 10.1186/s12876-021-01802-9

**Published:** 2021-05-13

**Authors:** Daniel W. Gunda, Elizabeth F. Mtui, Paulina M. Manyiri, David C. Majinge, Semvua B. Kilonzo, Humphrey D. Mazigo, Benson R. Kidenya

**Affiliations:** 1 Department of Internal Medicine, Catholic University of Health and Allied Sciences, 1464 Mwanza, Tanzania; 2 Department of Internal Medicine, Bugando Medical Centre, 1370 Mwanza, Tanzania; 3 Department of Parasitology, Catholic University of Health and Allied Sciences, 1464 Mwanza, Tanzania; 4 Department of Biochemistry and Molecular Biology, Catholic University of Health and Allied Sciences, 1464 Mwanza, Tanzania

**Keywords:** *Schistosoma mansoni*, Periportal fibrosis, Esophageal varices, APRI levels, PSDR levels Hepatitis B co-infection, Northwestern Tanzania, Resource-limited setting, Validation of APRI and PSDR use in *Schistosoma mansoni* PPF

## Abstract

**Background:**

*Schistosoma mansoni* related hepatic fibrosis is usually associated with hemodynamic alteration with increased mortality due to bleeding varices. The diagnosis of varices before bleeding imposes a big challenge in resource-limited countries using endoscopy. Published evidence on the utility of non-invasive clinical tools in predicting the presence of varices among patients with *S. mansoni* related periportal fibrosis is still inadequate including Aspartate to platelet ratio index (APRI) and Platelet to splenic diameter ratio (PSDR) levels. This study describes the determinants of portal varices and assesses the potential utility of the APRI and PSDR level in the discrimination of portal varices among patients with *S. mansoni* related periportal fibrosis (PPF).

**Methods:**

A case–control study using cross-sectional data was done among patients with *Schistosoma mansoni* related periportal fibrosis at Bugando Medical Centre, in Mwanza Tanzania. The derivation cohort included patients enrolled between 2015 and 2019 and the validation cohort included patients enrolled from 2019 till March 2021. Socio-demographic, laboratory, ultrasound, and upper digestive endoscopic information were analyzed using STATA 13. The prevalence and determinants of varices were determined by logistic regression. The sensitivity and specificity of independent factors were determined to assess their utility in discriminating the presence of portal varices in patients with PPF.

**Results:**

In total, 250 patients were included in the derivation cohort, 109 (43.6%; 95% CI 37.3–49.9) of them had varices. The odds of having varices were independently increased among patients with higher APRI levels than 1.51, (AOR: 5.8; 95% CI 3.1–11.1; *p* < 0.001) and PSDR levels that were lower than 5700 (AOR: 5.9; 95% CI 3.2–11.2; *p* < 0.001). Both APRI and PSDR levels had significantly high sensitivity and specificity in predicting the presence of esophageal varices. However, the combined values of APRI and PSDR had higher specificity than any of the two markers. Of the 200 patients in the validation cohort 94 (47.0%; 95% CI 40.0–54.2) had varices, the discriminative power of the final model and the predictive ability of both APRI, PSDR, and APRI-PSDR combined levels were highly maintained.

**Conclusions:**

This study indicates that varices are a common encounter among patients with *S. mansoni* related periportal fibrosis and it is independently associated with higher APRI and lower PSDR levels suggesting that these tools are potential discriminators of varices in this subgroup of patients. The reproducibility of these results should further be assessed longitudinally as potential non-invasive tools in selecting patients at high risk of having esophageal varices who could benefit from the targeted endoscopic intervention in a resource-limited setting like ours.

## Background

Chronic *Schistosoma mansoni* infection is a common cause of morbidity and mortality in a resource-limited setting where its transmission is ongoing. While 91.0% of the world's Schistosoma burden is found in Sub Saharan Africa (SSA), more than a 54million people are infected with *S. mansoni* in this region [1]. Morbidity reports indicate that over 20million people are chronically infected and *S. mansoni* related periportal fibrosis is reportedly the commonest and the most serious complication of chronic *Schistosoma mansoni* infection with high morbidity and mortality [2, 3].

About 0.2million deaths are reported annually in SSA due to complications of chronic *S. mansoni* infection. Heavy periportal oviposition occurs in *S. mansoni* infection with intense granuloma formation that ultimately graduates into periportal fibrosis (PPF), portal hypertension, and formation of esophageal varices [4, 5]. Field-based studies indicate that close to 50.0% of people who are chronically infected with *S. mansoni* have periportal fibrosis and in the hospital setting more than 70.0% of patients with periportal fibrosis have been found to have attendant portal varices [6–8].

However, these patients are often diagnosed late already with fatal bleeding varices, with mortality which may be as high as 29.0% even with the best available care [9, 10]. This is partly due to limited access to upper digestive endoscopy as a gold standard diagnostic modality in the most resource-limited setting. Endoscopy is of a maximal advantage when it is well-timed before incident bleeding [11] which would enable early identification of patients who could benefit from preventive treatment against bleeding varices and hence scale down the magnitude of subsequent mortality.

The formation of varices is linearly related to fibrosis and splenic size; but also inversely related to thrombocyte levels among others [12–15]. Out of these tests that are used in daily clinical practice some non-invasive tools have been developed including Aspartate aminotransferase (AST) to platelet count (PTC) ratio index (APRI) and Platelet to Splenic diameter ratio (PSDR) levels. The APRI levels have been used to assess the severity of fibrosis in patients with PPF with excellent sonographic and histological correlation [16, 17], however, there is still a paucity of studies describing the utility of APRI levels in the prediction of varices in patients with periportal fibrosis. The data on the use of PSDR levels in discriminating the presence of portal varices among patients with periportal fibrosis is still scarce as well when compared to patients with liver cirrhosis [12, 13, 18].

This study was designed to assess the utility of the APRI and PSDR levels in the prediction of varices among patients with periportal fibrosis in a Schistosoma endemic area of Tanzania. This information is clinically important in maximizing the sorting-out of patients at high risk of having varices and who could benefit from further interventions to mitigate the impact of late diagnosis.

## Methods

A case–control study using cross-sectional data was done among patients with PPF between 2015 and 2019 at Bugando Medical Centre (BMC) as a derivation cohort. A minimum sample size of 207 patients was estimated from the Lisle-Kish formula for cross-sectional studies, assuming 16.0% of patients with PPF had varices [19] with an allowable error of 0.05 at a 95%confidence interval (CI). The validation cohort included patients enrolled from 2019 onwards. The diagnosis of PPF was made sonographically as done previously [20], after informed consent. Seriously ill and pregnant patients were excluded. Sonographically, details on portal vein diameter (PVD), splenic diameters (SPD), and the presence of ascites were documented. All participants also underwent a test for active *S. mansoni* infection either by Urine Circulating Cathodic Antigen (CCA) or stool Kato Katz (KK). Hepatitis B surface antigen (HBsAg), liver injury (AST and ALT), and Complete blood count (CBC) was also done among others. Finally, all patients underwent upper digestive endoscopy to assess the presence of esophageal varices, praziquantel (PZQ) was given twice a year, Propranolol was added if the participants had small varices and band ligation for those with large varices.

Patients with PPF were serially enrolled until the sample size was reached. The information on research interest including patients’ socio-demographic data, clinical presentation like abdominal distension, hematemesis, and melaena, ultrasound (UTS) details; test results for *Schistosoma mansoni*, CBC, AST, ALT, serum Albumin (ALB), and upper digestive endoscopy results were included in analysis. Data were computerized using Epi data version 3.1 (Epidata DK. Denmark, EpiData Association) and STATA version 13 (Stata Corp LP, college station, TX) was used for analysis. Continuous variables were summarized as medians with interquartile range (IQR) and categorical variables as proportions with percentages.

Aspartate (AST) to platelet count (PTC) ratio index (APRI) and Platelet (PTC) to splenic diameter (SPD) were calculated as done previously [21, 22]. The presence of varices was expressed as a percentage with 95% Confidence Interval (CI) and its correlates were assessed. Based on earlier data and our own experience, socio-demographic factors, level of fibrosis (APRI values), markers of decompensation (ascites, serum albumin), and platelet to splenic diameter ratio (PSDR) [4, 12, 13, 23–25] were assessed for the association. The odds ratio (OR) with 95%CI was calculated by logistic regression to assess the degree of association between the various factors and the presence of esophageal varices. Factors with *p* < 0.2 on the univariate model were included in the multivariate model and the level of significance was set at *p* < 0.05.

The goodness of fit for the final model was assessed subsequently [26]. The sensitivity and specificity of independent factors in the final logistic models were also assessed to determine their discriminative ability including the APRI levels, PSDR levels, and a combined APRI and PSDR value for both the derivation and validation cohorts. The Receiver Operating Characteristic (ROC) curves were used according to Hanley and McNeil's method to determine the cut points with the best sensitivity and specificity for continuous variables which were reported as proportions with 95% CI [27].

### Ethical clearance

The permission to conduct and publish the findings from this study was sought from the Catholic University of Health And Allied Sciences and Bugando Medical Centre joint ethical committee with an ethical clearance certificate number 907/2019. The patients’ information was handled by the researcher alone and their identifiers including names and registration numbers were not included in the final analysis to further conserve confidentiality.

## Results

### Socio-demographic and clinical characteristics of 250 patients in the derivation cohort

A total of 250 participants were analyzed, males made the majority, 180 (72.0%; 95% CI 65.9–77.5) with a male to female ratio of 2.6: 1. The median age was 41(IQR: 33–51) years and most of them, 215 (86.0%; 95% CI 81.1–90.1) were married. In total, 222 (88.8%; IQR: 84.2–92.4) participants had a positive test for *S. mansoni* and 44 (17.6%; IQR: 13.1–22.9) tested positive for hepatitis B. Ascites was found in 155 (62.0%; 95% CI 55.7–68.0) participants, and both dilated portal veins and splenomegaly were common with median measurements in centimeters of 1.5 (IQR: 1.4–1.9) and 17 (IQR: 15–18) respectively (Table 1).

**Table 1:** Socio-demographic and clinical characteristics of the study cohorts

Variable	Derivation cohort (N = 250)		Validation cohort (N = 200)	
	Freq	% (95% CI)/median(IQR)	Freq	% (95% CI)/median(IQR)
SEX				
Male	180	72.0 (65.9–77.5)	145	72.5 (65.7–78.5)
Female	70	28.0 (22.5–34.1)	55	27.5 (21.4–34.2)
Age (Years)	250	41 [33–51]	200	40 [33–49]
Married				
Yes	215	86.0 (81.1–90.1)	168	84.0 (78.1–88.8)
No	35	14.0 (10.0–18.9)	32	16 (11.1–21.8)
Laboratory				
AST(U/L)	250	39.8 [34–56]	200	40.5 [34–58]
ALT(U/L)	250	32.5 [19–54]	200	32.5 [20.5–53.8]
PTC (*10^3^/µL)	250	99 [70–158]	200	100 [66–170]
*S. mansoni*+	222	88.8 (84.2–92.4)	180	90 (85.0–93.8)
HBsAg+	44	17.6 (13.1–22.9)	36	18 (12.9–24.0)
UTS abdomen				
Ascites	155	62.0 (55.7–68.0)	130	65.0 (58.0–71.6)
PVD (cm)	250	1.5 [1.4–1.7]	200	1.5 [1.4–1.7]
SPD (cm)	250	17 [15–18]	200	17 [16–19]
Endoscopy				
Varices present	109	43.6 (37.4–49.9)	94	47 (40.0–54.2)
Varices absent	141	56.3 (5.0–62.6)	106	53 (45.8–60.0)

ALT: alanine aminotransferase; AST: aspartate aminotransferase; HGB: hemoglobin; HBV: hepatitis B Virus; IQR: interquartile range; PTC: platelet counts; PVD: portal vein diameter; SPD: Splenic diameter

### Prediction of varices in the derivation cohort of 250 patients with periportal fibrosis

**Fig. 1:**
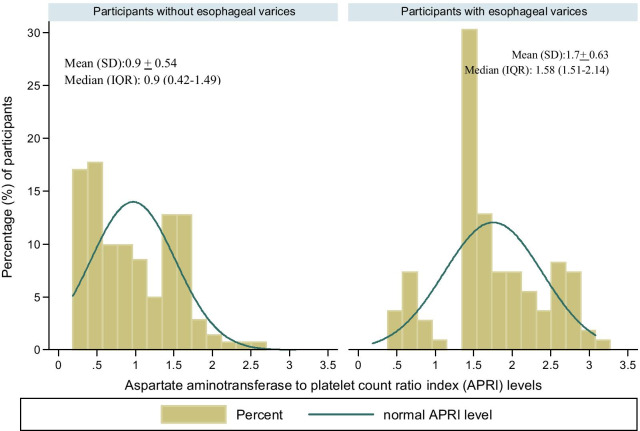
Distribution of Aspartate aminotransferase to platelet ratio index by varices

**Fig. 2:**
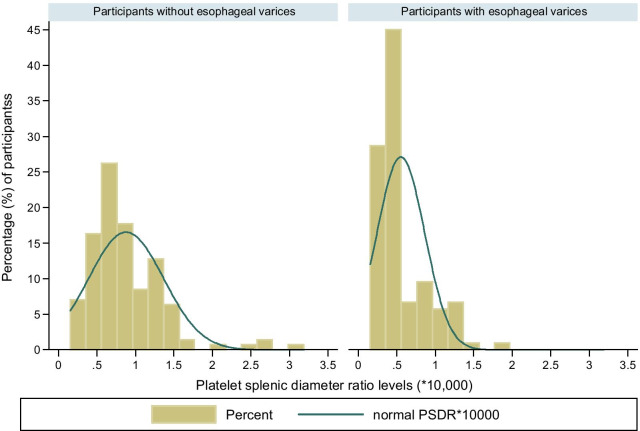
Distribution of Platelet to splenic diameter ratio levels by the presence of varices

**Fig. 3:**
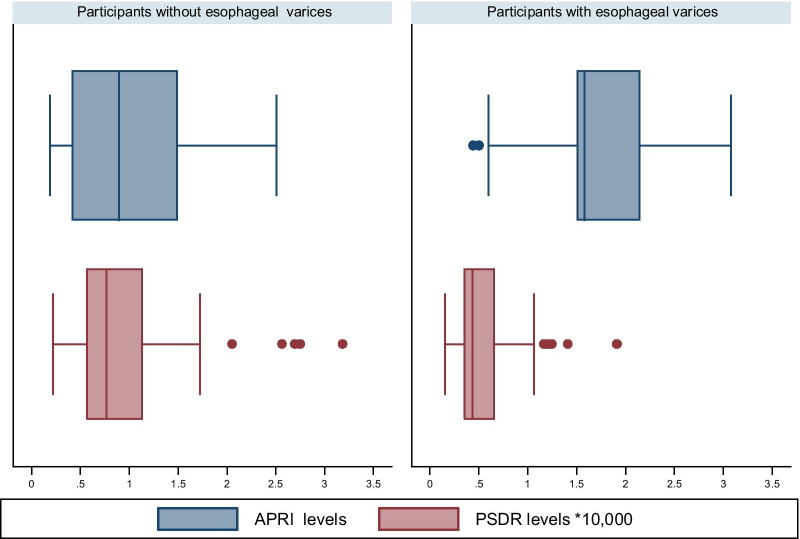
Box plot distribution of APRI and PSDR levels by the presence of varices

In this study, a total of 109 (43.6%; 95% CI 37.3–0.49.9) participants were found to have esophageal varices. The distribution of varices by APRI levels indicated that APRI levels were positively correlated to the development of varices (Table 2) where patients with esophageal varices were more likely to have higher APRI levels (Fig. 1, 3) as compared to their variceal negative counterparts. The PSDR levels, on the other hand, were inversely related to the presence of portal varices (Fig. 2, 3). On the multivariate model the odds of having varices were independently increased among patients with higher APRI levels than 15.1, (AOR: 5.8; 95% CI 3.1–11.1; *p* =  < 0.001) and PSDR levels that were lower than 5700 (AOR: 5.9; 95% CI 3.2–11.2; *p* < 0.001). Active *S. mansoni* and having ascites had a non-significant positive association with the presence of varices with *p* value > 0.05 (Table 3).

**Table 2:** Distribution of esophageal varices by APRI levels among 250 participants

Varices present	Aspartate aminotransferase to platelet ratio index		Total (n, %)
	< 1.5 (n, %)	> 1.5 (n, %)	
No (n, %)	113 (80.1)	28 (19.9)	141 (100.0)
Yes (n, %)	19 (17.4)	90 (82.6)	109 (100.0)
Total (n, %)	132 (52.8)	118 (47.2)	250 (100.0)
Pearson chi2(1) = 97.0091 Pr < 0.001			

APRI: aspartate aminotransferase to platelet ratio index; CI: confidence interval; OR: odds ratio, n: number

**Table 3:** Factors associated with varices among 250 patients with periportal fibrosis

Variable	Esophageal varices present		Un adjusted		Adjusted	
No, (n = 141)	Yes, (n = 109)	OR (95%CI)	P-value	OR (95%CI)	P-value	
Age, years*	42 [35–55]	39 [33–47]	0.9 (0.8–1.0)	0.169	1.0 (0.9–1.01)	0.498
Sex						
Female	39 ( 27.7)	31 (28.4)	1.0		-	-
Male	102 (72.3)	78 ( 71.6)	1.0 (0.5–1.7)	0.892	-	-
Married						
No	17 (12.1)	18 (16.5)	1.0		-	-
Yes	124 (87.9)	91 (83.5)	0.7 (0.3–1.4)	0.316	-	-
Alcohol use						
No	63 (44.7)	64 ( 58.7)	1.0			
Yes	78 (55.3)	45 ( 41.3)	0.6 (0.3–0.9)	0.028	0.6 (0.3–1.1)	0.109
*S. mansoni* test						
Negative	21 (14.9)	7 ( 6.4)	1.0			
Positive	120 (85.1)	102 (93.6)	2.5 (1.0–6.2)	0.040	2.4 (0.9–7.0)	0.095
Hepatitis B test						
Negative	116 (82.3)	90 (82.6)	1.0		-	-
Positive	25 (17.7)	19 (17.4)	0.9 (0.5–1.8)	0.951	-	-
APRI levels*						
< 1.51	114 (80.8)	37 (33.9)	1.0			
> 1.51	27 (19.2)	72 (66.1)	8.2 (4.6–14.6)	< 0.001	5.8 (3.1–11.1)	< 0.001
PCSDR levels**						
> 5700	106 (75.2)	39 (26.6)	1.0			
< 5700	35 (24.8)	80 (73.4)	8.3 (4.7–14.7)	< 0.001	5.9 (3.2–11.2)	< 0.001
PVD (CM)		1.5 [1.4–1.7]	1.5 [1.3–1.7]	0.9 (0.7–1.2)	0.858	
SPD (CM)	17 [15–18.3]	17 [16–18]	1.0 (0.9–1.1)	0.467		
Ascites present						
No	62 ( 44.0)	33 (30.3)	1.0			
Yes	79 ( 56.0)	76 (69.7)	1.8 (1.1–3.1)	0.028	1.3 (0.7–2.5)	0.469

^*^APRI levels are positively related to variceal formation, 1.51 is the best cut point,(AUC: 0.8259); by ROC curve; **PSDR levels are inversely related, 5700 is the best cut point by ROC curve (AUC: 0.624); ALT: Alanine aminotransferase; APRI: Aspartate aminotransferase-platelet index; AUC: area under the curve; HBsAg: Hepatitis B surface antigen; PVD: Portal vein diameter; ROC: receiver operating characteristic; SCHBCI: Schistosoma mansoni-Hepatitis B co-infection; SPD: Splenic diameter

**Fig. 4:**
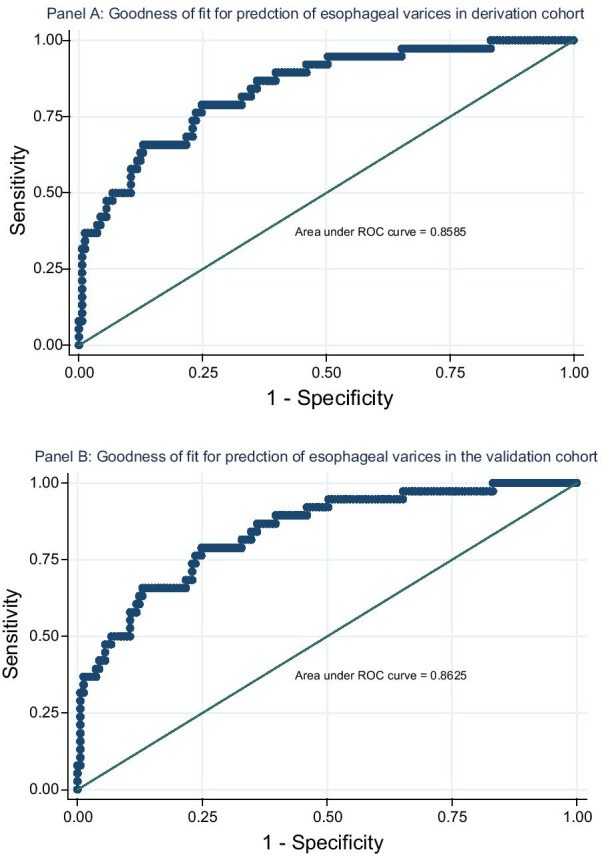
Receiver operating characteristic curve of the final model for prediction of varices

The assessment for the good of fitness of the final model did not demonstrate any gross lack of fit (Area under ROC curve: 0.8585; *p* = 0.314) (Fig. 4, panel A). The assessment for discriminative ability indicated that higher APRI levels, (cut point: 1.51) had both higher sensitivity, (82.5%; 95% CI 74.1–89.2) and specificity (80.1%; 95% CI 72.6–86.3) as compared to PSDR levels which also had acceptably good predictive ability (sensitivity: 73.4%; 95% CI 64.1–81.4; specificity: 75.2% 95% CI 67.2–82.1) at a cut point of 5700 in discriminating varices among patients with PPF. The combined APRI and PSDR values had a sensitivity and specificity of 54.1% (95% CI 44.3–63.7) and 94.3% (95% CI 89.1–97.5) respectively in predicting esophageal varices (Table 4).

**Table 4:** Predictive values of APRI, PSDR, and combined APRI-PSDR levels for portal varices

Variable	Cut point	Derivation cohort (N = 250)			Validation cohort (N = 200)		
		ROC	Sense (95% CI)	Spec (95% CI)	ROC	Sense (95% CI)	Spec (95%CI)
APRI	1.51	0.8259	82.5 (74.1–89.2)	80.1 (72.6–86.3)	0.8117	84.0 (75.0–91.0)	72.0 (62–80.0)
PSDR	5700	0.6471	73.4 (64.1–81.4)	75.2 (67.2–82.1)	0.7431	74.5 (64.4–83.0)	73.0 (63.1–81.0)
APRI-PSDR	NA	NA	54.1(44.3–63.7)	94.3 (89.1–97.5)	NA	71.3 (61.0–80.1)	71.0 (61.1–79.1)

APRI: Aspartate aminotransferase-platelet index; CI: confidence interval; NA: not applicable; PSDR: Platelet splenic diameter ratio; ROC: receiver operating curve; APRI-PSDR: combined APRI and PSDR value

### The validation of APRI and PSDR in a cohort of 200 patients with periportal fibrosis

The validation cohort includes 200 patients who are already enrolled in an ongoing 3 years PhD program at the gastroenterology and hepatology unit at Bugando medical center which started late in 2019. The population was comparable in major demographic and clinical characteristics (Table 1). Of the 200 participants in this validation cohort, 94(47.0%; 95% CI 40.0–54.2) had esophageal varices. The final multivariate logistic model maintained an excellent discrimination power, (Area under ROC curve: 0.8625; *p* = 0.1490) (Fig. 4, panel B). Similarly, at similar cut points, the sensitivity and specificity of both APRI levels, PSDR levels, and the combined APRI-PSDR levels were highly maintained in discriminating presence of esophageal varices (Table 4).

## Discussion

The objective of this study was to determine the prevalence and correlates of esophageal varices and assess its potential discriminators among patients with *S. mansoni* related periportal fibrosis. In this study, a total of 109 (43.6%) participants with periportal fibrosis were found to have esophageal varices, which were more likely to occur among patients with higher APRI levels and those with PSDR levels lower than 5700. The APRI and PSDR levels were both significantly sensitive and specific in predicting the presence of esophageal varices in this subgroup of patients.

The prevalence of varices in this study is similar to an earlier report of 45.0% from Uganda[28]and 47.0% reported from Sudan [29]. On the contrary, the prevalence of varices in this study is lower than what was reported earlier in Sudan, (43.6 vs. 67.0%) [30] and Saudi, (43.6 vs. 72.0%) among patients with PPF [8]. However, the current prevalence is higher than the prevalence of 16.0% reported recently from Sudan [19]. The differences in the prevalence of varices in these studies could partly be due to the difference in the severity of liver fibrosis among studied participants since portal varices have been reported to have a linear relation with fibrosis level [14].

In this study, age, alcohol use, active *S. mansoni*, presence of ascites, APRI, and PSDR levels were assessed for their independent association with esophageal varices in the final model, and the prediction ability of factors with the independent association was further determined by calculating their sensitivity and specificity. Active *S. mansoni* was previously reported to have an independent association with the presence of varices in a study done by Awilly et al. among patients with upper digestive tract bleeding [31]. In our study, the presence of active *S. mansoni* infection had only a non-significant positive correlation with the presence of varices, (AOR: 2.4; IQR: 0.9–7.0; *p* = 0.095).

A positive correlation between the presence of portal varices and advanced fibrosis by ultrasound has been described previously [4, 32, 33], in turn, some studies have reported a positive correlation between liver fibrosis determined by ultrasound with the APRI levels [16, 34]. The current finding that the portal varices in patients with PPF were independently common in patients with higher APRI levels suggests that APRI levels can be used to select patients at high risk of having varices in areas with limited services. This correlation suggests that varices develop in advanced PPF which in turn is associated with liver dysfunction and reduced thrombocyte count [35–37]. In the current study, we similarly observed a significant proportion of patients with elevated serum aspartate aminotransferase levels (ALT) and thrombocytopenia as summarized in Table 1.

A combination of AST and PTC into the APRI score in this study has suggested that besides the prediction of fibrosis severity [16, 34], this noninvasive tool can potentially be used in discriminating the presence of varices among patients with PPF. In this study, APRI levels had a sensitivity and specificity of 82.5 and 80.1% respectively at a cut point of 1.51 (area under ROC: 0.8259). The utility of this tool in predicting the presence of varices has been reported previously among patients with liver cirrhosis with results that are comparable to the findings of our current study among patients with *S.mansoni* related PPF (sensitivity:64.7–81.5%; specificity: 60–72.7% [38, 39].

The evidence on the utility of PSDR in the prediction of portal varices among patients with PPF is gradually growing. In the available body of literature, PSRD has been reported to have a sensitivity and specificity of 33.3–100% and 66.0–92.0% respectively as reported by authors from Saud with sensitivity: 100% (95% CI 89–100) and specificity: 92% (95% CI 62–99) [13]; China (sensitivity: 85.3% (95% CI 76.5–91.7%); specificity: 83.0% (95% CI 75.7–88.8%) [25] and Sudan (sensitivity: 33.3%; specificity: 66% [12]. The results of our study are consistent and falling within the reported ranges (sensitivity: 73.4%; 95% CI 64.1–81.4; specificity: 75.2%; 95% CI 67.2–82.1).Otherwise, the observed differences in the reported predictive values could partly be due to the small number of participants studied in previous studies (i.e. 43–109) and the difference in cut-off points [12, 13, 25].

In this study, the assessment of APRI and PSDR as a combined value in the discriminating portal varices indicated that the two parameters used together significantly increase the specificity to about 95% which is higher than any of the two parameters when used alone. However, with a lower sensitivity compared to either of the two parameters. Though this has not been reported before, these findings suggest that the combined value may potentially be useful in selecting patients with PPF who are more likely to have no varices and thus excluded from immediate endoscopic evaluation.

The current study is liable to some limitations; including the fact that this is a single-center study, its results may not be generalizable. But also there was no report of fibrosis grading by ultrasound as done in other studies, and fibrosis wasn’t confirmed by liver biopsy. None of our patients also underwent computed tomography as done in other studies. But also with the use of cross-sectional data, the temporal relationship between the outcome and exposure variables is difficult to ascertain. However, even with these limitations, the results from this study are important, especially in resource-limited settings where the burden of Schistosoma morbidity is high with serious resource restriction.

## Conclusions

This study indicates that varices are a common encounter among patients with *S. mansoni* related periportal fibrosis and it is independently associated with higher APRI and lower PSDR levels. The current results suggest that these tools may potentially be useful in the selection of patients at high risk of having varices for targeted endoscopic intervention in resource-limited settings. The reproducibility of these results should further be assessed longitudinally as potential non-invasive tools in selecting patients at high risk of having esophageal varices who could benefit from the targeted endoscopic intervention in a resource-limited setting like ours.

## Abbreviations

ALB: Albumin
ALT: Alanine aminotransferase
AOR: Adjusted odds ratio
AST: Aspartate aminotransferase
BMC: Bugando Medical Centre
CBC: Completer blood count
CCA: Circulating cathodic antigen
CUHAS: Catholic University of Health and Allied Sciences
HGB: Hemoglobin
HBV: Hepatitis B virus
HBsAg: Hepatitis B surface antigen
IQR: Interquartile range
KK: Kato Katz
PPF: Periportal fibrosis
PTC: Platelet counts
PVD: Portal vein diameter
PZQ: Praziquantel
ROC: Receiver operating characteristic
SCHBCI: Schistosoma mansoni-Hepatitis B co-infection
SSA: Sub Saharan Africa
SPD: Splenic diameter
UTS: Ultrasound
WHO: World Health Organization
